# Glycerol Improves Intracerebral Hemorrhagic Brain Injury and Associated Kidney Dysfunction in Rats

**DOI:** 10.3390/antiox10040623

**Published:** 2021-04-19

**Authors:** Cheng-Yi Chang, Ping-Ho Pan, Jian-Ri Li, Yen-Chuan Ou, Su-Lan Liao, Wen-Ying Chen, Yu-Hsiang Kuan, Chun-Jung Chen

**Affiliations:** 1 Department of Surgery, Feng Yuan Hospital, Taichung City 420, Taiwan; c.y.chang.ns@gmail.com; 2 Department of Veterinary Medicine, National Chung Hsing University, Taichung City 402, Taiwan; pph.pgi@gmail.com (P.-H.P.); wychen@dragon.nchu.edu.tw (W.-Y.C.); 3 Department of Pediatrics, Tungs’ Taichung Metro Harbor Hospital, Taichung City 435, Taiwan; 4 Division of Urology, Taichung Veterans General Hospital, Taichung City 407, Taiwan; fisherfishli@yahoo.com.tw; 5 Department of Nursing, HungKuang University, Taichung City 433, Taiwan; 6 Department of Urology, Tungs’ Taichung Metro Harbor Hospital, Taichung City 435, Taiwan; ycou228@gmail.com; 7 Department of Medical Research, Taichung Veterans General Hospital, Taichung City 407, Taiwan; slliao@vghtc.gov.tw; 8 Department of Pharmacology, Chung Shan Medical University, Taichung City 402, Taiwan; kuanyh@csmu.edu.tw; 9 Department of Medical Laboratory Science and Biotechnology, China Medical University, Taichung City 404, Taiwan

**Keywords:** brain edema, hemorrhagic stroke, intracranial pressure, kidney dysfunction, stress hormones

## Abstract

In stroke patients, the development of acute kidney injury (AKI) is closely linked with worse outcomes and increased mortality. In this study, the interplay between post-stroke and AKI and treatment options was investigated in a rodent model of hemorrhagic stroke. Intrastriatal collagenase injection for 24 h caused neurological deficits, hematoma formation, brain edema, apoptosis, blood–brain barrier disruption, oxidative stress, and neuroinflammation in Sprague Dawley rats. Elevation of serum blood urea nitrogen, serum creatinine, urine cytokine-induced neutrophil chemoattractant-1, and urine Malondialdehyde, as well as moderate histological abnormality in the kidney near the glomerulus, indicated evidence of kidney dysfunction. The accumulation of podocalyxin DNA in urine further suggested a detachment of podocytes and structural deterioration of the glomerulus. Circulating levels of stress hormones, such as epinephrine, norepinephrine, corticosterone, and angiotensin II were elevated in rats with intracerebral hemorrhage. Osmotic agent glycerol held promising effects in alleviating post-stroke brain injury and kidney dysfunction. Although the detailed protective mechanisms of glycerol have yet to be determined, the intrastriatal collagenase injection hemorrhagic stroke model in rats allowed us to demonstrate the functional and structural integrity of glomerulus are targets that are vulnerable to post-stroke injury and stress hormones could be surrogates of remote communications.

## 1. Introduction

Stroke is a leading cause of neurological disability and mortality worldwide, resulting in huge public health and socioeconomic burden. There are two main types of stroke, ischemic and hemorrhagic stroke, and approximately 87% of stroke patients are ischemic [1]. Chronic kidney disease (CKD) has long been recognized as a risk factor for stroke. Conversely, stroke may lead to kidney dysfunction, and acute kidney injury (AKI) adversely impacts patient outcomes [2,3,4,5]. Thus, a better understanding of post-stroke AKI and identifying intervention strategies may help provide options for ameliorating post-stroke disease progression.

AKI is characterized by kidney dysfunction resulting in disturbance of electrolytes, acid–base, and homeostasis of fluids with a clinical spectrum ranging from mild, asymptomatic injury to severe injury. Serum and urine biomarkers, as well as estimated glomerular filtration rate (eGFR), have been validated for the early detection of AKI. Serum cystatin C level, but not creatinine, blood urea nitrogen (BUN), β2-microglobulin, and eGFR in the intensive care unit have been demonstrated to be important biomarker for predicting AKI and mortality in stroke patients [3]. Therefore, the identification of alternative biomarkers for the early detection of stroke-associated AKI is of interest for therapeutic treatments.

Oxidative stress, neuroinflammation, blood–brain barrier (BBB) disruption, and apoptosis have substantial roles in secondary brain injury after an ischemic and hemorrhagic stroke. The latter includes intracerebral hemorrhage (ICH) and subarachnoid hemorrhage (SAH) [6,7,8,9]. Additionally, alteration of the sympathetic nervous system, hypothalamic pituitary axis, and renin–angiotensin–aldosterone system may occur and impact stroke disease outcome involving oxidative stress and neuroinflammation [10,11,12]. The generation and expansion of hematoma are closely linked to hemorrhagic stroke neurological deficits. Hematoma and derived products act as neurotoxins and mainly contribute to edema formation and brain injury [13]. Clinically, brain edema represents a deteriorative complication after hemorrhagic stroke and may lead to higher intracranial pressure and a worse outcome [14]. Osmotherapeutic agents are useful adjuncts to reduce neuroinflammation following hemorrhagic stroke [15]. Besides its effects on the brain, over-activation of the sympathetic nerve system, hypothalamic–pituitary axis, and renin–angiotensin–aldosterone system impairs kidney function, thereby contributing to developing AKI [12,16,17]. This highlights the possibility that osmotic agents may have an ameliorating effect on stroke-associated kidney dysfunction.

Osmotic agents have been used to treat brain edema in stroke patients. Although its effects on the noninfarcted hemisphere and large hemispheric infarction remain controversial, glycerol possesses the ability to decrease brain edema and intracranial pressure [18,19,20]. Mannitol and hypertonic saline further reduce ICH brain injury and neuroinflammation [15]. To investigate whether osmotic agents are potentially capable of alleviating post-stroke brain injury and associated kidney dysfunction, the effects of glycerol were evaluated in a rodent model of stroke. Patients with hemorrhagic stroke suffer from a worse outcome than those with ischemic stroke, and hemorrhagic transformation is a common complication of ischemic stroke, which is exacerbated by thrombolytic therapy [21]. Therefore, an ICH animal model was established using Sprague-Dawley to investigate the aforementioned phenomena.

## 2. Materials and Methods

### 2.1. Experimental Allocation and ICH Induction

Adult male Sprague-Dawley rats (200–230 g) were purchased from BioLASCO (Taipei, Taiwan) and were kept in conventional cages with free access to food and water. Rats were allocated to four groups: sham with saline (n = 32); ICH with saline (n = 32); sham with glycerol (n = 32); ICH with glycerol (n = 32). Under anesthesia with isoflurane (2–4%), the rat’s head was fixed in a stereotactic apparatus, and stereotactic surgery was performed following the relevant study [22]. ICH was induced by injection of type IV collagenase (0.3 U/2 μL saline) through a Hamilton syringe at the coordinates corresponding to the striatum (3 mm lateral to the midline, 0.2 mm posterior to bregma, depth 6 mm below the surface of the skull). After injection, the syringe was remained for an additional 3 min to minimize the leakage of collagenase. The burr holes were then sealed with bone wax. Sham-operated rats received the same stereotactic surgical processes and saline injection. The experimental protocols adhered to the Institute’s guidelines and were approved by the Institutional Animal Care and Use Committee of Taichung Veterans General Hospital (IACUC approval code: La-100859, IACUC approval date: 11 November 2011). Glycerol at a dose of 6 mL/kg (10% glycerol) or an equal volume of saline was intraperitoneally injected into rats 30 min after surgery. The dose of glycerol was performed according to a previously described study with modifications [23]. All rats were euthanized for analyses 24 h after surgery.

### 2.2. Morphological Examination

After euthanasia, the brains (n = 8 per group) were quickly removed, chilled in cold phosphate-buffered saline (PBS), and 2 mm coronal slices were cut using a tissue splicer. Seven sections were photographed, and all visible areas with hematoma were delineated. The percentage of the marked area was calculated.

### 2.3. Neurological Evaluation

To evaluate sensorimotor performance (n = 8 per group), a modified six-point neurological deficit severity scoring criteria was applied according to our previously reported study [24]. The scoring criteria in the neurological evaluation were as follows: 0, no neurological deficit; 1, difficulty in fully extending the left forepaw; 2, unable to extend the left forepaw; 3, mild circling to the left; 4, severe cycling to the left; and 5, falling to the left.

### 2.4. Brain Edema Evaluation

After euthanasia, the brains (n = 8 per group) were quickly removed and separated into contralateral and ipsilateral hemispheres to isolate the striatum. The dissected contralateral and ipsilateral striatal tissues were dried in an oven at 110 °C for 24 h. The water content was calculated by the wet/dry weight method [24]. Data are expressed as the subtraction of ipsilateral content with contralateral content in the same rat.

### 2.5. Caspase 3 Activity Assay

After euthanasia, the brains (n = 8 per group) were quickly removed and separated into contralateral and ipsilateral hemispheres to isolate the striatum. The dissected ipsilateral striatal tissues were subjected to the measurement of caspase 3 activity using a caspase-3 colorimetric assay kit (BioVision, Mountain View, CA, USA).

### 2.6. Evans Blue Extravasation Assay

Three hours prior to the end of the study, Evans blue (4%, 1 mL/kg) was injected into the rats via the tail vein. After euthanasia, rats (n = 8 per group) were perfused with heparinized saline. The dissected ipsilateral striatal tissues were weighed, homogenized in PBS (500 μL), and centrifuged. The obtained supernatants were mixed with trichloroacetic acid (500 μL, 100%) and stand overnight at 4 °C. After centrifugation at 12,000 rpm at 4 °C for 10 min, the supernatants were collected and subjected to the measurement of Evans blue content using a spectrophotometer (absorbance at 620 nm). The contents of Evans blue were calculated using a standard solution.

### 2.7. Urine, Blood, and Tissue Sample Collection

Prior to sacrifice for analyses, rats were housed in metabolic cages for 12 h for the collection of urine samples. At the end of the study, rats were euthanized, and the blood samples were withdrawn from the left femoral artery. Tissues of the kidney and brain were rapidly dissected and stored in liquid nitrogen or were soaked in formalin until analyses could be performed.

### 2.8. Histological Examination

After euthanasia, the left kidney (n = 8 per group) was quickly removed, fixed in 4% buffered formaldehyde, and embedded in paraffin. Sections (4 μm) were then deparaffinized, rehydrated, and stained with Hematoxylin/Eosin (H&E) according to standard procedures. Digitalized images were captured at 200X and 400X magnification using a light microscope equipped with a digital camera (Nikon, ECLIPSE, 50i, Tokyo, Japan).

### 2.9. Biochemical Analyses

The serum levels of BUN and creatinine were measured using automated, standardized procedures (Roche Hitachi 917/747, Mannheim, Germany). The levels of kidney injury molecule-1 (KIM-1; R&D Systems, Minneapolis, MN, USA), cytokine-induced neutrophil chemoattractant-1 (CINC-1; R&D Systems, Minneapolis, MN, USA), neutrophil gelatinase-associated lipocalin (NGAL, R&D Systems, Minneapolis, MN, USA), corticosterone (BioVendor, Germany), norepinephrine (LDN, Nordhorn, Germany), epinephrine (LDN, Nordhorn, Germany), and angiotensin II (Ang II, R&D Systems, Minneapolis, MN, USA) were determined through enzyme-linked immunosorbent assay (ELISA) kits according to the manufacturer’s instructions.

### 2.10. Lipid Peroxidation Product Measurement

Levels of lipid peroxidation product (n = 8 per group) were measured using a TBARS assay kit (Cayman Chemical, Ann Arbor, MI, USA) according to the manufacturer’s instructions. Data are expressed as Malondialdehyde (MDA) equivalents.

### 2.11. Measurement of Glutathione (GSH)

Levels of GSH (n = 8 per group) in dissected ipsilateral striatal tissues and kidney cortical tissues were measured using a Glutathione Assay Kit (Cayman Chemical, Ann Arbor, MI, USA) according to the manufacturer’s instructions.

### 2.12. Measurement of Tumor Necrosis Factor-α (TNF-α) and Interleukin-1β (IL-1β)

After euthanasia, the brains (n = 8 per group) were quickly removed and separated into contralateral and ipsilateral hemispheres to isolate the striatum. The dissected ipsilateral striatal tissues were subjected to the measurement of TNF-α and IL-1β content using ELISA (R&D Systems, Minneapolis, MN, USA).

### 2.13. Western Blot

After euthanasia, the brains (n = 8 per group) were quickly removed and separated into contralateral and ipsilateral hemispheres to isolate the striatum. The dissected ipsilateral striatal tissues were subjected to the extraction of proteins (tissue protein extraction reagents, Pierce Biotechnology, Rockford, IL, USA) and conduction of a standardized SDS–PAGE and PVDF membrane transfer. After incubation with 5% skim milk for 30 min, the specific proteins on the membranes were recognized with the corresponding antibodies, including Matrix Metalloproteinase-9 (MMP-9, mouse monoclonal antibody, 1:1000), MMP-2 (mouse monoclonal antibody, 1:1000), Zonula Occludens-1 (ZO-1, rat monoclonal antibody, 1:1000), cluster of differentiation 68 (CD68, mouse monoclonal antibody, 1:1000), myeloperoxidase (MPO, mouse monoclonal antibody, 1:500), p65 (rabbit polyclonal antibody, 1:1000), phospho-p65 (mouse monoclonal antibody, 1:500), and glyceraldehyde 3-phosphate dehydrogenase (GAPDH, mouse monoclonal antibody, 1:3000) (Santa Cruz Biotechnology, Santa Cruz, CA, USA). Proteins on the membranes were visualized by the sequential incubation with horseradish peroxidase-conjugated IgG and enhanced chemiluminescence Western blotting reagents. The chemiluminescent blots were scanned using the G:BOX mini multi fluorescence and chemiluminescence imaging system (Syngene, Frederick, MD, USA). The intensity of immunoreactive signals was quantitated by ImageJ software (National Institute of Health, Bethesda, MD, USA).

### 2.14. Zymography Assay

After euthanasia, the brains (n = 8 per group) were quickly removed and separated into contralateral and ipsilateral hemispheres to isolate the striatum. The dissected ipsilateral striatal tissues were subjected to the extraction of proteins (tissue protein extraction reagents, Pierce Biotechnology, Rockford, IL, USA) and conduction of a standardized SDS–PAGE (8%). After separation, the gels were washed twice for 30 min with 2.5% Triton X-100 and then incubated in buffer (25 mM Tris, 150 mM NaCl, 10 mM CaCl_2_, 0.2% Brij-35, pH 7.5) overnight at 37 °C. Afterward, the gels were stained with Coomassie brilliant blue R-250 (0.2%). The intensities of visualized bands were quantitated by ImageJ software (National Institute of Health, Bethesda, MD, USA).

### 2.15. Electrophoretic Mobility Shift Assay (EMSA)

After euthanasia, the brains (n = 8 per group) were quickly removed and separated into contralateral and ipsilateral hemispheres to isolate the striatum. The dissected ipsilateral striatal tissues were subjected to the extraction of nuclear proteins (NE-PER nuclear and cytoplasmic extraction kit, Thermo Fisher Scientific, Waltham, MA, USA) and conduction of EMSA (LightShift^TM^ chemiluminescent EMSA Kit, Thermo Fisher Scientific, Waltham, MA, USA) according to the manufacturer’s instructions. The sequences of oligonucleotide were: NF-κB, 5′-AGTTGAGGGGACTTTCCCAGGC. The intensity of the bands was analyzed by ImageJ software (National Institute of Health, Bethesda, MD, USA).

### 2.16. Semi-Quantitative Polymerase Chain Reaction (PCR)

The pooled urine samples (n = 8 per group) were centrifuged at 2000 rpm for 5 min at 4 °C. Total DNA was extracted from the cell pellets using a DNA isolation kit (Abcam, Cambridge, UK). The PCR consisted of 30 cycles of reaction (denaturation at 95 °C for 45 s, annealing at 60 °C for 30 s, and extension at 72 °C for 1 min) and a final extension at 72 °C for 10 min. The amplified DNA products were separated by 1.5% agarose gel electrophoresis and stained with ethidium bromide. The intensity of the bands was analyzed by ImageJ software (National Institute of Health, Bethesda, MD, USA). The oligonucleotides for PCR were: 5′-GCAGGGCTTTGAACCTCTTG and 5′-GCTCTGTGACACTCGGATTT for podocalyxin; 5′-AGATCCACAACGGATACATT and 5′-TCCCTCAAGATTGTCAGCAA for GAPDH.

### 2.17. Statistical Analysis

Experimental data were analyzed by SPSS software and expressed as mean values ± standard deviation. All data were first analyzed by one-way or two-way analysis of variance (ANOVA), and the statistical differences were determined by Dunnett post hoc analysis. The level of significance was set at *p* < 0.05.

## 3. Results

### 3.1. Glycerol Alleviated Hemorrhagic Stroke Brain Injury

**Figure 1:**
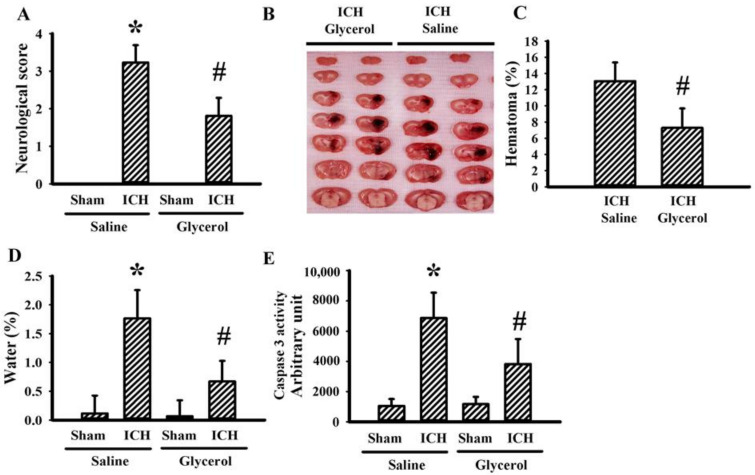
Glycerol alleviated hemorrhagic stroke brain injury. ICH and sham rats were intraperitoneally injected with saline or glycerol and housed for an additional 24 h. (**A**) Neurological deficits were evaluated by neurological score. (**B**) Representative photographs show histological examination of hematoma. (**C**) The percentage of hematoma in the ipsilateral striatum is depicted. (**D**) The water content differences between ipsilateral and contralateral striatum were measured. (**E**) Proteins were extracted from the ipsilateral striatal tissues and subjected to the measurement of caspase 3 activity. * *p* < 0.05 vs. sham/saline and # *p* < 0.05 vs. ICH/saline, n = 8.

Intrastriatal collagenase injection for 24 h caused neurological deficits (Figure 1A), brain hematoma formation (Figure 1B,C), brain edema (Figure 1D), and caspase 3 activations (Figure 1E) in rats. A dose of intraperitoneal glycerol injection alleviated brain injury in hemorrhagic stroke rats (Figure 1). The findings suggest a beneficial effect of glycerol against hemorrhagic stroke brain injury.

### 3.2. Glycerol Alleviated Hemorrhagic Stroke BBB Disruption

**Figure 2:**
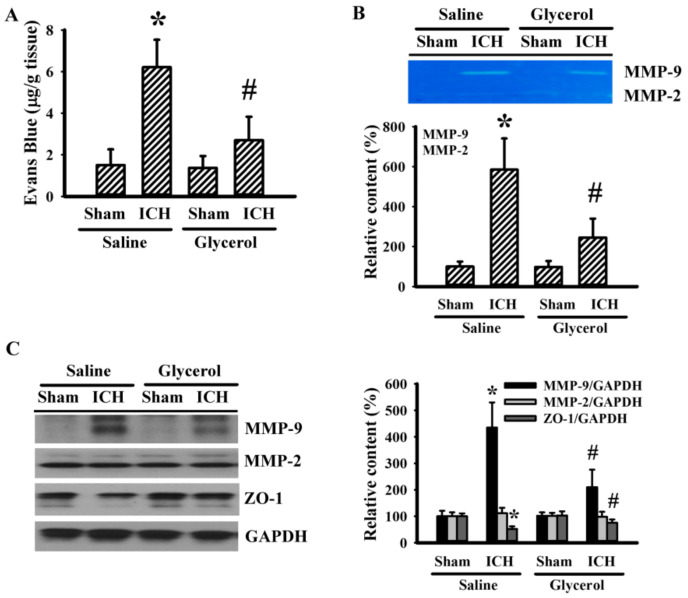
Glycerol alleviated hemorrhagic stroke BBB disruption. ICH and sham rats were intraperitoneally injected with saline or glycerol and housed for an additional 24 h. (**A**) The contents of Evans blue in the ipsilateral striatal tissues were measured. (**B**) Proteins were extracted from the ipsilateral striatal tissues and subjected to zymography assay. Representative gels and the quantitative data are shown. (**C**) Proteins were extracted from the ipsilateral striatal tissues and subjected to Western blot assay with indicated antibodies. Representative blots and the quantitative data are shown. Specific protein content was normalized with GAPDH. * *p* < 0.05 vs. sham/saline and # *p* < 0.05 vs. ICH/saline, n = 8.

ICH is associated with the disruption of BBB [7,8,25]. Parameters of BBB integrity were examined. An apparent Evans blue extravasation (Figure 2A) was observed in the ipsilateral striatal tissues of hemorrhagic stroke rats, and the elevation was paralleled with enhanced MMP-9 activity (Figure 2B), increased MMP-9 protein expression (Figure 2C), and reduced tight junction ZO-1 protein expression (Figure 2C). However, the change of MMP-2 was not of significance (Figure 2C). Glycerol injection alleviated all the changes in hemorrhagic stroke rats (Figure 2). In other words, ICH rats display increased cerebrovascular permeability, and the disruption of the BBB could be alleviated by glycerol.

### 3.3. Glycerol Alleviated Hemorrhagic Stroke Oxidative Stress and Neuroinflammation

**Figure 3:**
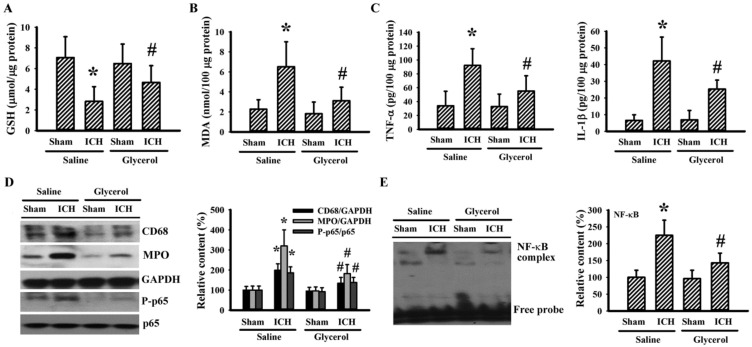
Glycerol alleviated hemorrhagic stroke oxidative stress and neuroinflammation. ICH and sham rats were intraperitoneally injected with saline or glycerol and housed for an additional 24 h. The contents of GSH (**A**) and MDA (**B**) in the ipsilateral striatal tissues were measured. (**C**) Proteins were extracted from the ipsilateral striatal tissues and subjected to ELISA for the measurement of TNF-α and IL-1β. (**D**) Proteins were extracted from the ipsilateral striatal tissues and subjected to Western blot assay with indicated antibodies. (**E**) Nuclear proteins were extracted from the ipsilateral striatal tissues and subjected to EMSA for the measurement of NF-κB DNA-binding activity. Representative blots and the quantitative data are shown. Specific protein content was normalized with the corresponding total protein or GAPDH. * *p* < 0.05 vs. sham/saline and # *p* < 0.05 vs. ICH/saline, n = 8.

Oxidative stress and neuroinflammation are pivotal to the pathogenesis of hemorrhagic stroke. Agents show antioxidant and/or anti-inflammatory potential displaying neuroprotective effects against hemorrhagic stroke [8,26,27]. ICH caused a decreased content of GSH (Figure 3A) and increased content of lipid peroxidation product MDA (Figure 3B), TNF-α and IL-1β inflammatory cytokine (Figure 3C), macrophage/microglia-related CD68 protein, neutrophil-related MPO protein, NF-κB p65 protein phosphorylation (Figure 3D), and NF-κB DNA-binding activity (Figure 3E) in ipsilateral striatal tissues. ICH-induced alterations could be reversed by glycerol (Figure 3). The findings indicate a resolution of oxidative stress and neuroinflammation in hemorrhagic stroke rats by glycerol.

### 3.4. Glycerol Alleviated Hemorrhagic Stroke-Associated Kidney Dysfunction

**Figure 4:**
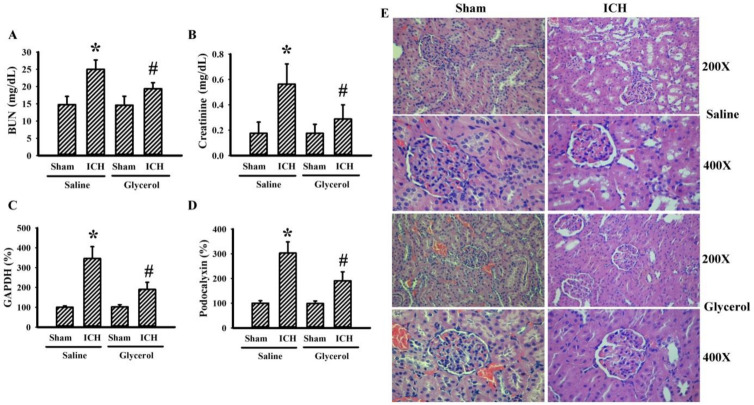
Glycerol alleviated hemorrhagic stroke-associated kidney dysfunction. ICH and sham rats were intraperitoneally injected with saline or glycerol and housed for an additional 24 h. Serum samples were subjected to the measurement of BUN (**A**) and creatinine (**B**). Urine samples were subjected to DNA isolation and PCR for the measurement of GAPDH (**C**) and podocalyxin (**D**) DNA content. Paraffin sections of kidney tissues were subjected to histological staining with H&E (**E**). Representative photomicrographs showed one of eight independent rats. * *p* < 0.05 vs. sham/saline and # *p* < 0.05 vs. ICH/saline, n = 8.

**Figure 5:**
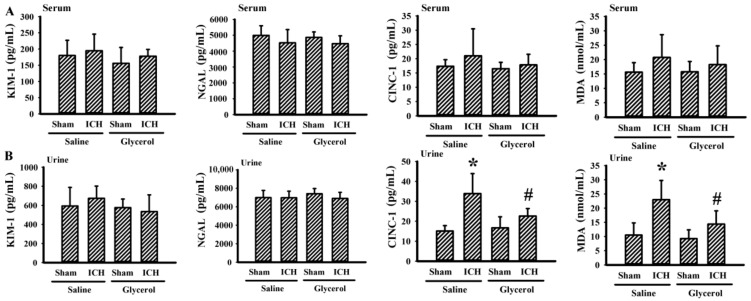
Glycerol alleviated urine biomarkers in hemorrhagic stroke rats. ICH and sham rats were intraperitoneally injected with saline or glycerol and housed for an additional 24 h. Serum (**A**) and urine (**B**) samples were subjected to ELISA for the measurement of KIM-1, NGAL, and CINC-1 as well as to TBARS assay for the measurement of MDA. * *p* < 0.05 vs. sham/saline and # *p* < 0.05 vs. ICH/saline, n = 8.

**Figure 6:**
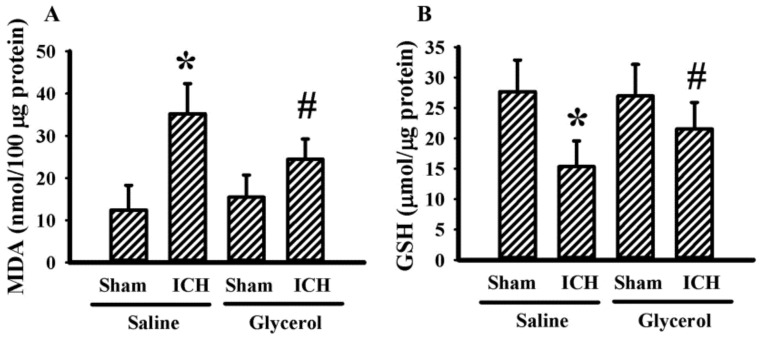
Glycerol alleviated kidney oxidative stress in hemorrhagic stroke rats. ICH and sham rats were intraperitoneally injected with saline or glycerol and housed for an additional 24 h. Kidney cortical tissues were isolated and subjected to the measurement of MDA (**A**) and GSH (**B**) * *p* < 0.05 vs. sham/saline and # *p* < 0.05 vs. ICH/saline, n = 8.

To identify any kidney dysfunction in hemorrhagic stroke rats, several biochemical and histological examinations centered on kidney structural and functional integrity were conducted. Hemorrhagic stroke rats had an increased level of serum BUN (Figure 4A) and creatinine (Figure 4B), as well as a level of urinary GAPDH (Figure 4C) and podocalyxin (Figure 4D) DNA content. Moreover, histological examination with H&E further revealed a mild dilation of the Bowman’s capsule, tubular dilation, and cast formation (Figure 4E). The biochemical and histological changes in the hemorrhagic stroke rats could be alleviated by glycerol injection (Figure 4). Hemorrhagic stroke-associated kidney dysfunction was further validated by serum and urinary KIM-1, NGAL, CINC-1, and MDA, early biomarkers of AKI [3]. There was no remarkable change in the measurements except urinary CINC-1 and urinary MDA. Hemorrhagic stroke rats had increased urine levels of CINC-1 and MDA, and the increments could be alleviated by glycerol injection (Figure 5). Moreover, an increase in MDA content (Figure 6A) and a reduction in GSH content (Figure 6B) were found in kidney cortical tissues of hemorrhagic stroke rats. The changes could be alleviated by glycerol injection (Figure 6). Current findings indicate a concurrent kidney dysfunction in hemorrhagic stroke rats and an alleviating effect of glycerol.

### 3.5. Glycerol Alleviated Stress Hormones in Hemorrhagic Stroke Rats

**Figure 7:**
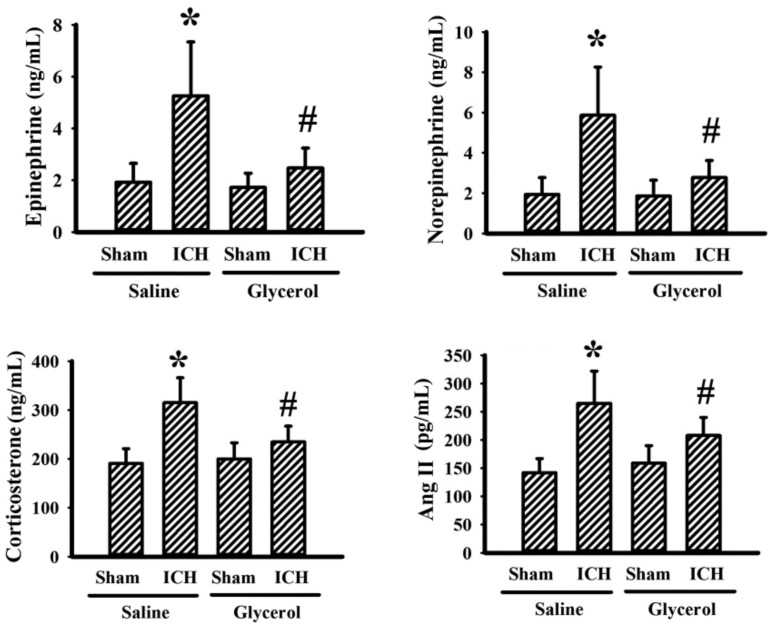
Glycerol alleviated stress hormones in hemorrhagic stroke rats. ICH and sham rats were intraperitoneally injected with saline or glycerol and housed for an additional 24 h. Serum samples were subjected to ELISA for the measurement of epinephrine, norepinephrine, corticosterone, and Ang II. * *p* < 0.05 vs. sham/saline and # *p* < 0.05 vs. ICH/saline, n = 8.

Stress hormones are adaptive molecules functioning in tissue homeostasis, while they also cause tissue/organ injury [10,11,12,16,17]. Hemorrhagic stroke rats exhibited increased levels of serum epinephrine, norepinephrine, corticosterone, and Ang II. Glycerol injection alleviated elevated stress hormones in hemorrhagic stroke rats (Figure 7). The findings suggest that ICH could increase stress hormone production, and the increments could be alleviated by glycerol.

## 4. Discussion

CKD is recognized as a risk factor for stroke with deteriorative effects by itself and is commonly accompanied by hypertension, hypercholesterolemia, and diabetes mellitus [2]. Having an episode of AKI after stroke is associated with worse outcomes and increased in-hospital mortality. Clinically, the reported rate of AKI after stroke varies widely with a range of 0.82% to 30.18% [2,3,5,28,29,30]. Khatri et al. [29] report that stroke patients with ICH in intensive care units and wards are more likely than patients with ischemia to develop AKI. In an intrastriatal collagenase injection hemorrhagic stroke model, we demonstrated that hemorrhagic stroke rats developed kidney dysfunction, as evidenced by elevated serum levels of BUN and creatinine. The elevations reached a peak at 24 h after collagenase injection and returned to normal ranges 72 h later (data not shown). Although the worst effects of AKI on stroke brain injury have not been investigated, the current data clearly demonstrate an occurrence of kidney dysfunction after a hemorrhagic stroke. However, the effects of an AKI in a rat model of ischemic stroke were not evaluated in this study.

To date, several serum and urine biomarkers have been validated to detect kidney injury at various stages. KIM-1, NGAL, CINC-1, lipid peroxidation product, cystatin C, BUN, creatinine, IL-18, β2-microglobulin, and eGFR are common biomarkers [3,31,32,33]. Among stroke patients in intensive care units, serum cystatin C is an important biomarker for predicting AKI and in-hospital mortality [3]. Elevated levels of serum BUN, serum creatinine, urinary CINC-1, and urinary MDA were found in intrastriatal collagenase injection hemorrhagic stroke rats. KIM-1, NGAL, serum CINC-1, and serum MDA appeared to be less strongly correlated. CINC-1 is a counterpart of human IL-8 family members, mediating inflammatory reactions by attracting neutrophils. Disease progression of AKI is associated well with renal neutrophil infiltration, cytokine expression, and oxidative stress [34]. The current study suggests that urinary CINC-1 and MDA are sensitive biomarkers than their serum counterparts in hemorrhagic stroke rats.

Besides serum and urinary biomarkers, the hemorrhagic stroke affected kidney structural integrity, particularly near the glomerulus. The glomerulus is a globular structure, and the filtering unit of the kidney is surrounded by the Bowman’s capsule. Podocytes are specialized epithelial cells located outside the glomerular basement membrane, wrapping glomerular capillaries to form the filtration barrier. Podocyte detachment and its urine accumulation represent alternative biomarkers of kidney injury [35]. The presence of GAPDH DNA in urine samples of intrastriatal collagenase injection hemorrhagic stroke rats reflected a leakage of cells from the kidneys. Our data further indicated the detached cells could be podocytes due to the successful detection of podocalyxin DNA, a sialoglycoprotein of podocytes [35]. The alteration of hemodynamics or kidney oxidative stress is considered a possible cause of podocyte detachment [31,35]. The elevation of renal and urinary MDA and reduction of renal GSH found in this study may highlight a potential involvement of oxidative stress in kidney structural changes.

Following a stroke, various changes and deterioration in function, structure, and remote communications occur. Among the remote communications, the sympathetic nervous system, hypothalamic pituitary axis, and renin–angiotensin–aldosterone system are pivotal in regulating renal blood flow, free radical generation, inflammatory response, and glomerular filtration [12,16,17,31]. Intrastriatal collagenase injection hemorrhagic stroke rats showed an elevated circulating level of epinephrine, norepinephrine, corticosterone, and Ang II. Therefore, prolonged elevation of stress hormones after stroke may lead to kidney dysfunction observed in this study. The roles and importance of epinephrine, norepinephrine, corticosterone and Ang II in hemorrhagic stroke-associated kidney dysfunction can be investigated by introducing pharmacological antagonists. However, their involvements were not addressed in the current study. Collagenase has been implicated in the deterioration of blood vessels and kidney glomerulus [36]. Despite the small dose and intrastriatal injection, the remote effects of collagenase on kidney dysfunction could not be totally excluded.

Usually, hemorrhagic stroke causes primary and secondary brain injury as well as the above-mentioned remote organ dysfunction. The mass effect of hematoma is the main cause of primary brain injury. In contrast, the extravasated blood components induce a wave of inflammatory and oxidative change leading to BBB disruption, edema, neuronal cell dysfunction/destruction, and other events of secondary brain injury. Agents or strategies intervening in hematoma expansion, oxidative stress, inflammation, BBB disruption, edema, apoptosis, or associated hemorrhagic stroke changes have promising effects in the prevention and treatment of hemorrhagic stroke brain injury and complications [6,7,8,9,13,15,25,26,27,37]. Brain edema and increased intracranial pressure severely threaten the disease progression and outcome of stroke patients [14]. In rodent models, short hypothermia was found to be effective at decreasing intracranial pressure after ischemic stroke [38]. Clinically, osmotic agents, such as mannitol and glycerol, are commonly used to treat increased intracranial pressure. Reduction of brain water content, enhancement of cerebrospinal fluid absorption, and increased cerebral blood flow are proposed beneficial mechanisms of osmotic agents [2,18,20]. Mannitol and hypertonic saline further reveal beneficial outcomes against hemorrhagic stroke brain injury and neuroinflammation [15]. The neurological deficit, hematoma formation, brain edema, BBB disruption, apoptosis, oxidative stress, and inflammation were demonstrated in the ipsilateral striatal tissues of ICH rats. A dose of intraperitoneal glycerol injection after stroke not only protected the brain from hemorrhagic injury but also improved accompanying kidney dysfunction. An NF-κB-dominant axis has been implicated in the expression and activation of macrophages/microglia, neutrophils, an inflammatory cytokine, and MMP after hemorrhagic stroke [7,8,25,27]. Consistent with the findings, data of NF-κB DNA-binding activity, p65 protein phosphorylation, CD68, MPO, and MMP-9 protein expression, and TNF-α and IL-1β production demonstrated the occurrence of NF-κB axis in ICH rats and could be intervened by glycerol. We further identified a concurrent change in BBB disruption, MMP-9 activation, and ZO-1 degradation in ICH rats, implying a contribution of the MMP-9/ZO-1 pathway in hemorrhagic stroke cerebrovascular permeability change. Although using glycerol in treating brain edema remains controversial, the current rodent study provides evidence demonstrating its beneficial effects again post-stroke brain injury and kidney dysfunction. Despite the encouraging findings, there are some limitations in interpreting experimental data. Clinically, stroke patients have been prescribed glycerol as an infusion dose of 250 mL (10%) at intervals of 4–6 h [18,20]. Rats show no significant kidney injury upon treatment with intraperitoneal hypertonic glycerol solution injection (10 mL/kg, 50%) followed by 24 h water deprivation. However, there are signs of oxidative stress observed in plasma and the kidney [23]. In this study, glycerol at a dose of 6 mL/kg (10%) was administrated intraperitoneally 30 min after surgery. Therefore, further investigation is needed to determine the optimal therapeutic regimen and to monitor the criteria for the application of glycerol.

## 5. Conclusions

CKD has long been considered a risk factor for stroke, while AKI after stroke worsens patient outcomes. Although the deteriorative correlation has yet to be determined, the intrastriatal collagenase injection hemorrhagic stroke model in rats presented herein demonstrated the occurrence of kidney dysfunction after stroke onset. Data from histological, biochemical, and molecular studies identified the functional and structural integrity of the glomerulus are targets vulnerable to post-stroke injury, and stress hormones could be surrogates of remote communications. Although the osmotic agent glycerol held promising effects in alleviating post-stroke brain injury and kidney dysfunction demonstrated by this study, its specific targets of action and protective mechanisms had not been fully investigated. Therefore, the clinical translation of current rodent model studies warrants further investigation.

## Data Availability

No new data were created or analyzed in this study. Data sharing is not applicable to this article.
